# Maternal factors during pregnancy influencing maternal, fetal and childhood outcomes: Meet the Guest Editors

**DOI:** 10.1186/s12916-022-02294-4

**Published:** 2022-03-10

**Authors:** Louis Muglia, Stephen Tong, Susan Ozanne, Katrien Benhalima

**Affiliations:** 1 Burroughs Wellcome Fund, Research Triangle Park, NC USA; 2 Department of Pediatrics, Cincinnati Children’s Hospital Medical Center and University of Cincinnati College of Medicine, Cincinnati, OH USA; 3 Department of Obstetrics and Gynaecology, University of Melbourne and Mercy Perinatal, Mercy Hospital for Women, Melbourne, Australia; 4 Wellcome-MRC Institute of Metabolic Science and Medical Research Council Metabolic Diseases Unit, University of Cambridge, Addenbrooke’s Hospital, Cambridge, UK; 5 Department of Endocrinology, University Hospital Gasthuisberg, KU Leuven, Herestraat 49, 3000 Leuven, Belgium

## *What is the focus of your current research concern and what are the main problems that need addressing in the field* ?

### Louis J. Muglia, MD PhD

The focus of my research efforts over the last 25 years has been to try and answer the question: “What determines the timing for birth?” While being a fundamental issue in understanding normal reproduction, the topic has enormous public health relevance as preterm birth, defined as delivery at less than 37 weeks of completed maternal gestation, has been and remains the leading cause of infant mortality, and more recently is the leading cause of death in children under 5 years of age [1]. Because of our lack of mechanistic insight into human birth timing, no generally effective preventative or active treatment strategies currently exist. To gain a non-biased view into the pathways leading to human parturition, we have utilized human genetic and genomic approaches for genome-wide interrogation of the maternal and fetal genomes together with how environmental exposures shape pregnancy outcomes [2]. Some key issues that need to be addressed are centered on further elucidation of fundamental parturition control mechanisms in human pregnancy, how social determinants mediate the risk for preterm birth, identification of variants that determine genetic predisposition, and how environment interacts with genetic predisposition or leads to epigenetic changes that modify birth timing [3]. Additionally, how is fetal growth coordinated with the duration of gestation and defining when labor will initiate? With this information, rational approaches to preventative strategies and new therapeutics can be developed. I look forward to the manuscripts in this collection advancing our understanding and clinical impact.

### Katrien Benhalima, MD PhD

I am an endocrinologist and clinician-scientist with a research focus on how to screen for gestational diabetes mellitus (GDM) [4], to evaluate the influence of maternal factors such as depression and thyroid function on GDM, to search for better predictors of the long-term cardio-metabolic risk after delivery, and to develop prevention strategies to reduce the long-term risk. Women with a history of GDM are a particularly high-risk population to develop type 2 diabetes (T2DM) and cardiovascular diseases at a younger age [5]. Moreover, offspring of mothers with GDM are also at increased risk to become obese and develop T2DM as young adults [6]. Besides GDM, my research also focuses on the evaluation of new technologies to improve pregnancy outcomes in pregnant women with type 1 diabetes (T1DM). We are currently performing a large RCT to evaluate the efficacy and safety of closed-loop insulin delivery in T1DM pregnancy. Several evidence gaps persist in the domain of GDM and diabetes in pregnancy. For instance, more research is needed on how to prevent GDM, on whether treatment of GDM in early pregnancy is beneficial, on non-fasting biomarkers to screen for GDM, on new biomarkers to predict pregnancy complications in women with diabetes, on the use of new technologies to manage diabetes in pregnancy and on how to reduce the long-term metabolic risk in mothers and offspring with a history of GDM or diabetes. I hope to welcome many manuscripts in this collection focusing on the impact of GDM or diabetes on pregnancy outcomes and/or long-term postpartum metabolic risks.

### Susan Ozanne, BSc (Hons) PhD

I am a basic scientist who has worked in the field, now called the Developmental Origins of Health and Disease for over 25 years. Specifically, my research focusses on understanding the mechanisms by which suboptimal exposures during fetal life impact on our long-term risk of poor cardio-metabolic health, including increased risk of type 2 diabetes, cardiovascular disease, and obesity [7, 8]. My work has therefore incorporated studies at the physiological, cellular, and epigenetic level in both model systems and in humans. I passionately believe that by understanding the mechanisms by which such “programming” occurs, we gain insight into what may be the best interventions to improve the short- and long- term health of both the mother and child. I have addressed this in the context of both maternal undernutrition as well as maternal over-nutrition, with my most recent studies focusing on the effects of maternal obesity and gestational diabetes during pregnancy. This is an area of particular concern with over half of women in many populations now either overweight or obese when entering pregnancy and one in seven pregnancies globally affected by gestational diabetes. It is therefore critical that we act now to reduce/prevent transmission of poor cardio-metabolic health from mother to child. I look forward to this collection receiving many manuscripts on this topic which both advance our mechanistic understanding and lead to the identification of rationale intervention strategies with the potential to improve the health of at least two generations.

### Stephen Tong, MBBS PhD

I am a clinician-scientist (specialist obstetrician) with a keen focus on translational research—developing diagnostics and treatments for major pregnancy complications. One of my early concepts developed in the laboratory a decade ago is a new drug treatment for ectopic pregnancy. The idea has survived three early phase clinical trials and is just completing recruitment in a large multi-centre trial in the UK (GEM III trial). Over the past 8 years, my team established a drug screening pipeline to find drugs for preeclampsia [9]. This pipeline begins with laboratory evaluation of drug candidates where the most promising ones are put to the test in randomised clinical trials in South Africa (we have finished two trials, found metformin as a promising treatment, and planning the next trial [10]). I co-lead research to develop a diagnostic test to help women avoid stillbirth, and I lead a dynamic team using big data to explore the safety of common obstetric interventions and drugs given during pregnancy.

I am honored to work with three stellar guest editors on an exciting theme that resonates with me—maternal influences on fetal outcomes. The mother’s influence on the health of the pregnancies is all-pervading. To cite one example of many, the health of mum’s blood vessels as she enters pregnancy heavily dictates whether she will develop preeclampsia, a condition which risks’ fetal health. I am thrilled *BMC Medicine* has initiated this special issue which reflects the journal’s interest to publish papers from my field of obstetrics.
